# Coexistence of periorbital lichen planus pigmentosus and pemphigus vulgaris: Report of an unusual case and a rare association

**DOI:** 10.1002/ccr3.4480

**Published:** 2021-07-16

**Authors:** Fatemeh Mohaghegh, Zahra Talebzadeh, Mahsa Bahraminejad, Mina Rezaei

**Affiliations:** ^1^ Department of Dermatology Isfahan University of medical sciences Isfahan Iran; ^2^ Department of Dermatology Skin Diseases and Leishmaniasis Research Center School of Medicine Isfahan University of Medical Sciences Isfahan Iran; ^3^ School of Medicine Isfahan University of Medical Sciences Isfahan Iran

**Keywords:** coexistence, lichen planus pigmentosus, pemphigus vulgaris, periorbital lichen planus pigmentosus

## Abstract

Lichen planus pigmentosus is a rare variant of lichen planus with different patterns and manifestations. The coexistence of LPP and PV suggests that there might be a relationship between these two conditions in terms of immunologic mechanisms.

## Graphical abstract

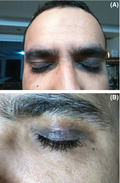

## 1 INTRODUCTION

Lichen planus pigmentosus (LPP) is a variant of lichen planus characterized by purple‐black macules and patches on sun‐exposed areas such as face. Here we report a rare case of periorbital lichen planus pigmentosus which was accompanied by pemphigus vulgaris shortly after we began treatment with topical steroid and calcineurin inhibitors.

Lichen planus is a chronic inflammatory disease causing cutaneous eruption and mucosal involvement. Lichen planus pigmentosus (LPP) is a rare variant of lichen planus characterized by dark brown or slate gray macules in sun‐exposed areas like face and neck, and it mostly occurs in middle‐aged dark‐skinned individuals.^1^ Only few cases of periorbital lichen planus pigmentosus have been reported previously.^2^, ^3^ It is a rare clinical manifestation of LPP which sometimes masquerades as racoon eye.^3^

Pemphigus vulgaris is an autoimmune disease destroying the keratinocyte connections causing vesicle. Recent studies have reported the associations between pemphigus vulgaris and other autoimmune conditions such as hypothyroidism, irritable bowel disease, type 1 diabetes, rheumatoid arthritis, and systemic lupus erythematous.^4^, ^5^ The coexistence of pemphigus vulgaris and oral lichen planus has been reported previously.^6^ A case report from 1987 has also reported the coexistence of general lichen planus and pemphigus vulgaris.^7^ Here we report a rare case of a patient presenting with periorbital pigmentation diagnosed with lichen planus pigmentosus which was later accompanied by pemphigus vulgaris.

## 2 CASE REPORT

**FIGURE 1:**
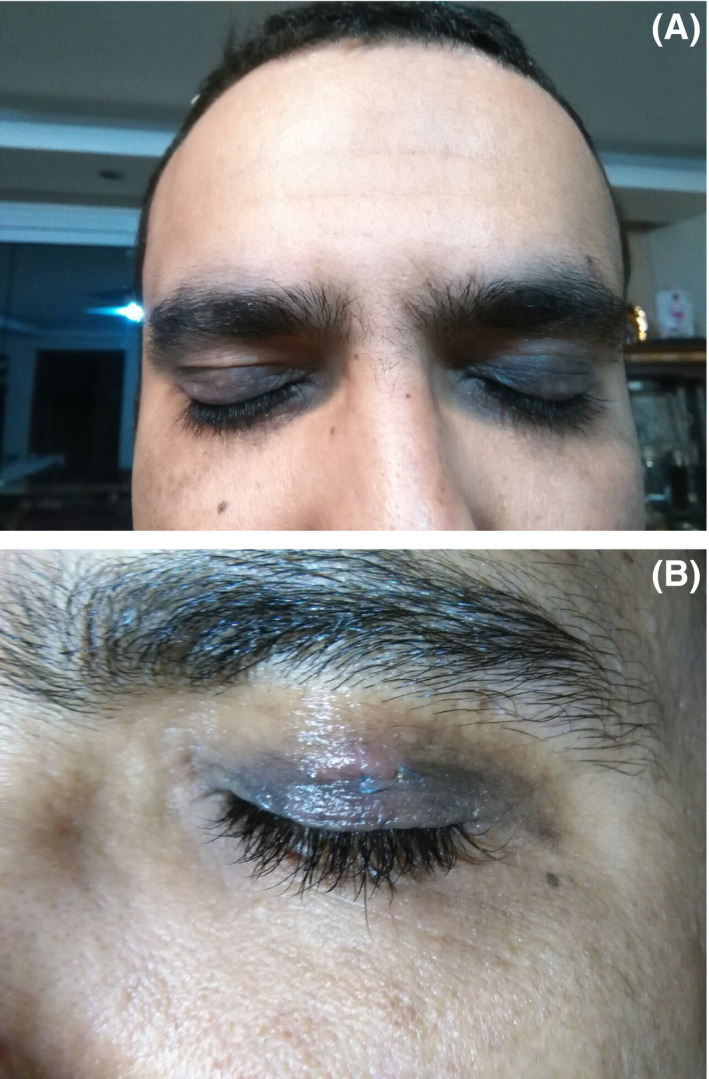
(A,B) purple‐black discoloration of lower and upper eyelids

A 39‐year‐old male patient presented to our dermatology department with the chief complaint of bilateral pigmentation and darkening of upper and lower eyelids for months. There was no history of associated trauma, sun exposure, drugs, and cosmetics. He had a history of keratoconus that was taken care of by hard lens under an ophthalmologist observation and grade one fatty liver. Physical examination revealed purple‐black discoloration of upper and lower eyelids and medial and lateral canthus of both eyes without ulceration and telangiectasia (Figure 1A,B).

**FIGURE 2:**
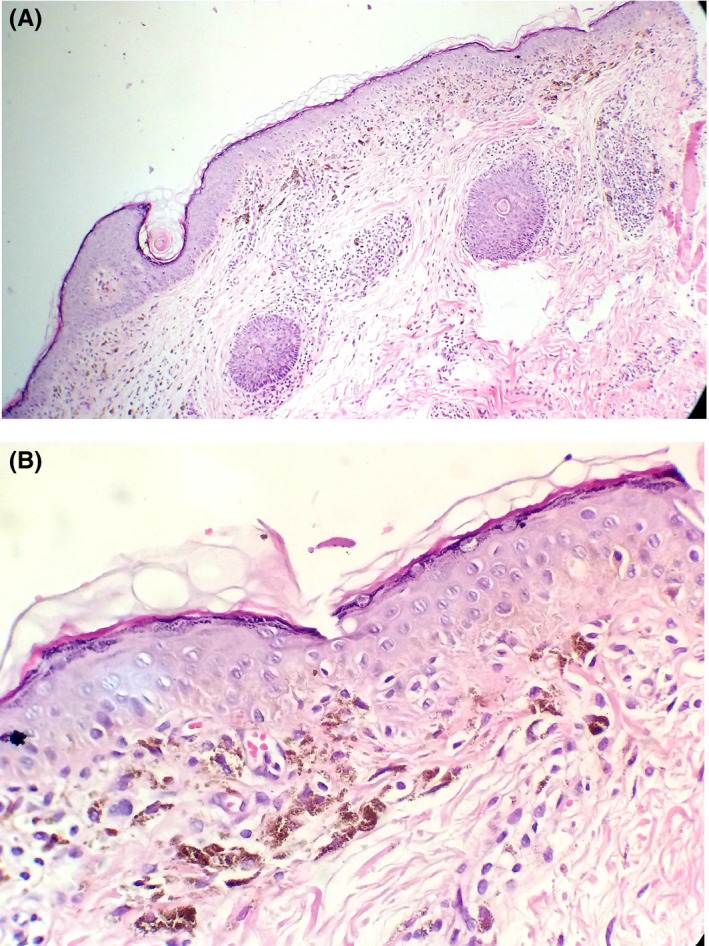
(A) Histopathology of palpebral lesion showing atrophy of the epidermis and melanin incontinence (H&E×100). (B) Focal basal cell layer degeneration, and few civatte bodies are noted in high power (H&E×400)

He had no pruritus. The primary diagnosis of lichen planus pigmentosus was confirmed by the biopsy results reporting epidermal atrophy, vacuolar degeneration, and obvious melanin incontinence. (Figure 2A,B).

**FIGURE 3:**
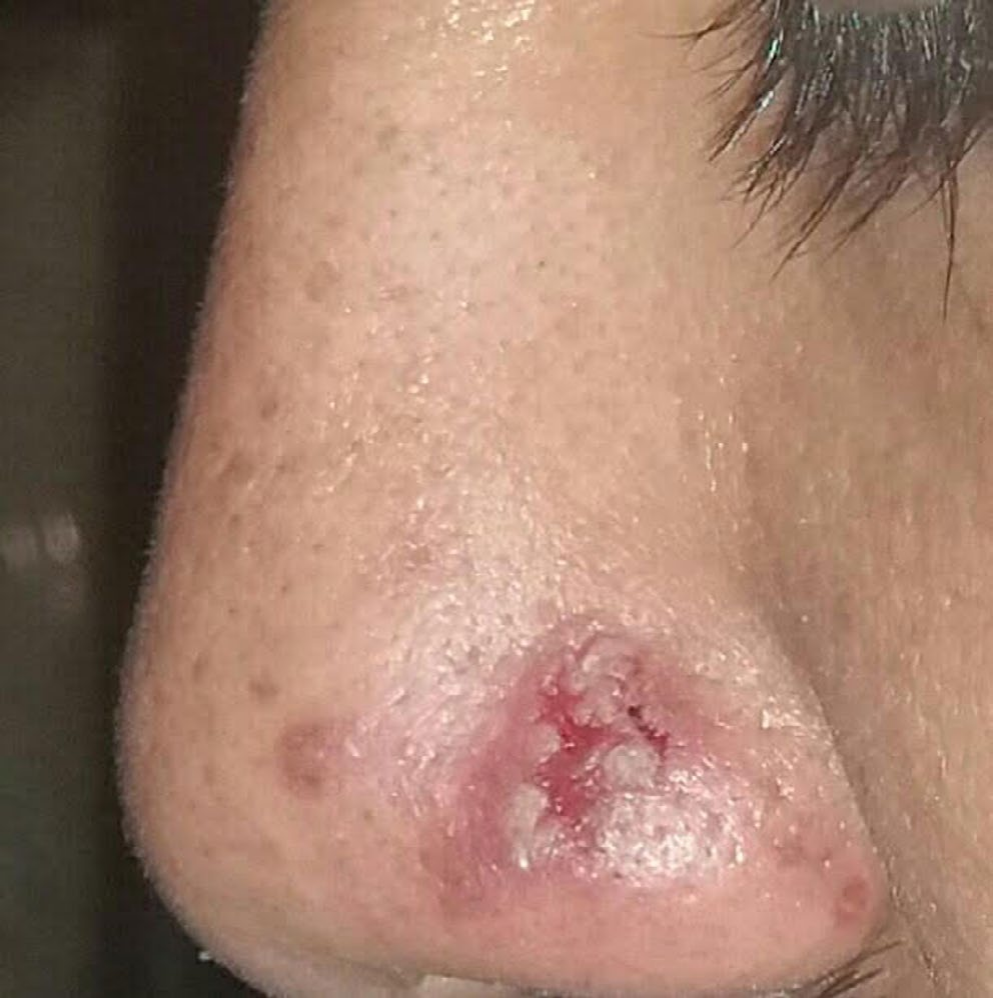
Nasal erosion

After consulting the ophthalmology department regarding his past medical history, we initiated treatment with topical ophthalmic betamethasone and topical calcineurin inhibitor (Pimecrolimus, Elidel [R]). Following 2 months of partial recovery, he complained of erosions on his nose and scalp, which did not respond to topical antibiotic therapy properly after 3 weeks (Figure 3). He also complained of bleeding while brushing his teeth. Two biopsy specimens of oral mucosa and nose erosion (from the advancing edge) were performed for routine histopathology, and one biopsy for direct immunofluorescent study was done on peripheral erythematous skin of nose erosion.

**FIGURE 4:**
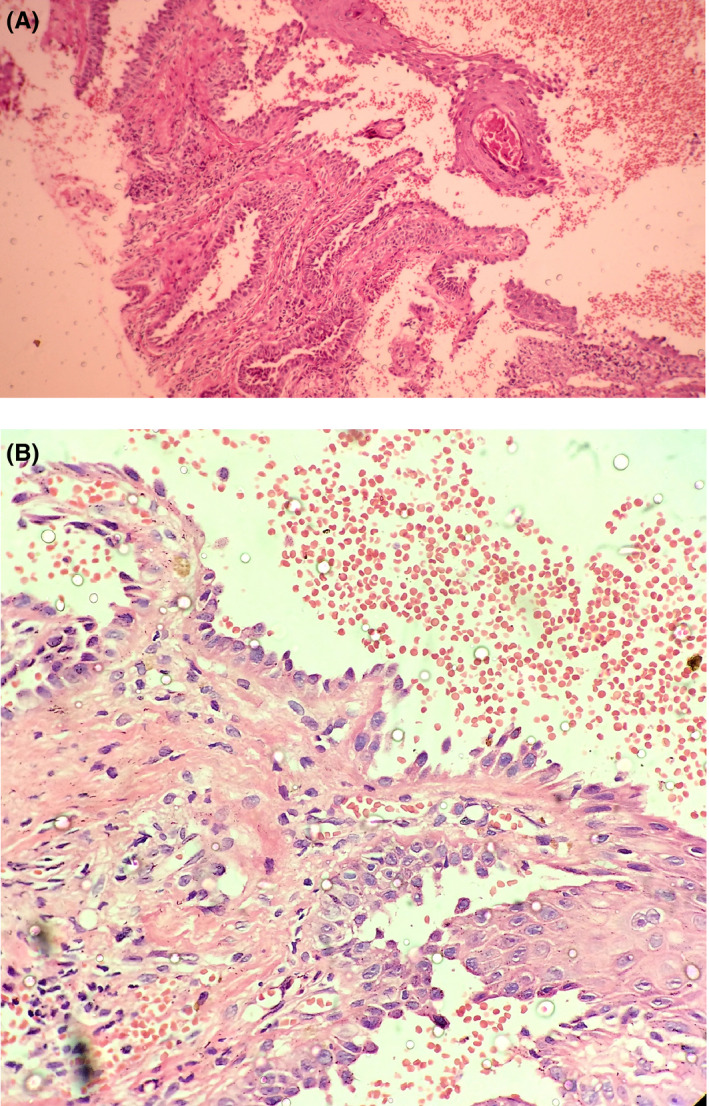
(A) Histopathology of nasal skin lesion showing suprabasal cleft (H&E×100). (B) High power shows thumb stone appearance. (H&E×400)

Microscopic examination of the oral lesion showed eosinophilic spongiosis that may occur in early pemphigus lesions. Histopathologic examination of nasal skin lesion exhibited suprabasilar clefting in epidermis with acantholytic cells in the split region. Basal cell layer had a characteristic “tombstone” appearance on the floor of the vesicle (Figure 4A,B). Direct immunofluorescence showed intercellular deposits of IgG and C3 in a “fish‐net” appearance. Indirect immunofluorescence was done on patient serum and both anti‐Dsg‐1 and anti‐Dsg‐3 antibody checked and anti‐Dsg‐3 antibody titers were high and reported positive (anti‐Dsg‐3: 1:320). A definite diagnosis of pemphigus vulgaris was made based on clinical, histopathological, and DIF findings.

We began 60 mg of daily prednisolone and tapered the dosage to 15 mg every other day within a year as the lesions started to recover. New lesions appeared on his nose and scalp afterward which were resistant to topical steroid and steroid injection. Finally, we continued the treatment with a daily dosage of 30 mg of prednisolone and 50 mg of azathioprine twice a day that led to full recovery of the skin lesions. Patient is currently under treatment, and new lesions have not appeared yet.

## 3 DISCUSSION

Lichen planus pigmentosus is an uncommon variant of lichen planus with an unknown etiology.^8^ It can be concomitant with other conditions such as hepatitis C infection, nephrotic syndrome, and head and neck cancers.^9^, ^10^, ^11^ Lichen planus pigmentosus is characterized by brown‐bluish macules on sun‐exposed parts of the body like face and neck, which can later progress to hyperpigmented patches.^12^, ^13^, ^14^ It has various patterns including diffuse, reticular, linear, perifollicular, blaschkoid, and zosteriform.^15^, ^16^, ^17^ Unlike lichen planus, Wickham striae and pruritus are often absent in lichen planus pigmentosus.^18^ Typical histopathologic findings in LPP include vacuolar degeneration of the basal cell layer with perivascular or lichenoid infiltrate, pigmentary incontinence, keratinocyte apoptosis, and superficial dermal melanophages.^8^

Periorbital linear lichen planus pigmentosus is an extremely rare condition. Bilateral periorbital involvement has been reported previously.^2^, ^3^ Considering the involvement of eyelids, most probable differential diagnoses would be dermal melanocytosis, post‐inflammatory hyperpigmentation secondary to atopic dermatitis, allergic contact dermatitis, Riehl's melanosis or pigmented cosmetic dermatitis (PCD), fixed drug eruption (FDE), and tear trough associated with aging.^5^ Treatment modalities for LPP include topical steroids, topical calcineurin inhibitors (pimecrolimus, tacrolimus), dapsone, and most importantly photoprotection.^2^

Pemphigus vulgaris is an autoimmune condition caused by circulating autoantibodies against keratinocyte cells that involves skin and mucosa. Previous studies have investigated coexistent diseases with pemphigus vulgaris and suggested that the prevalence of rheumatoid arthritis, type 1 diabetes, autoimmune thyroid disease, irritable bowel disease, and systemic lupus erythematous increases in patients with pemphigus vulgaris compared to the general population.^4^, ^5^ There are reports of coexistence of pemphigus vulgaris and pemphigus foliaceus with oral lichen planus.^19^ Balighi et al reported the coexistence of pemphigus vulgaris with oral lichen planus in which pemphigus lesions responded to rituximab but lichen planus lesions did not. This suggested that the pathology of these two conditions might be different despite all the other clinical and immunological similarities.^6^ Also, Lee et al^7^ reported a case of generalized lichen planus coexistent with pemphigus vulgaris in 1987. Lajevardi et al studied the role of autoantibodies against desmoglein‐1 and desmoglein‐3 in the pathogenesis of lichen planus, which revealed an increased level of anti‐Dsg‐3 antibody in erosive oral lichen planus.^20^ Also in another case report, Michele D Mignogna et al. reported the case of two female patients who after an initial diagnosis of oral lichen planus developed mucous membrane pemphigoid in a period ranging from 3 to 11 years. Data provided in that case report originated the hypothesis that epitope spreading phenomenon might be the underlying mechanism of this event.^21^

In this case, the simultaneous occurrence of LPP and PV was significant and suggests that there might be a relationship between these two conditions in terms of pathophysiology and etiology. Also, the coexistence of these two conditions could be an accidental finding as it is extremely rare. Further studies need to be done to investigate the possible relationship between LPP and PV.

## 4 CONCLUSION

Lichen planus pigmentosus is a rare variant of lichen planus with different patterns and manifestations. The coexistence of LPP and PV suggests that there might be a relationship between these two conditions in terms of immunologic mechanisms.

## CONFLICTS OF INTERESTS

The authors have no conflict of interest to declare.

## AUTHOR CONTRIBUTION

All the authors listed in the manuscript have participated actively and equally in presenting the case and providing the final version of the manuscript.

## ETHICAL APPROVAL

Written consent was taken from the patient for publishing the case report including the pictures. This case report was approved by the bioethics committee of Isfahan University of Medical Sciences.

## Data Availability

No dataset was generated or analyzed during this case report.

## References

[1] Law D , Vahdani K , Ashdown M , Garrott H , Ford R . Periorbital linear lichen planus pigmentosus—report of 2 cases and literature review. Can J Ophthal. 2018;54:e12‐e14. 30851786 10.1016/j.jcjo.2018.03.004

[2] Tiwary AK , Kumar P . Bilateral periorbital involvement localized to eyelids in lichen planus pigmentosus. Indian Dermatol Online J. 2018;9:58‐59. 29441303 10.4103/idoj.IDOJ_117_17 PMC5803947

[3] Arora AK , Kumaran MS , Saikia UN , Narang T . Lichen planus pigmentosus masquerading as ‘Raccoon eyes’. Pigment Int. 2016;3:114‐115.

[4] Parameswaran A , Attwood K , Sato R , Seiffert‐Sinha K , Sinha AA . Identification of a new disease cluster of pemphigus vulgaris with autoimmune thyroid disease, rheumatoid arthritis, and type I diabetes. Br J Dermatol. 2014;172:729‐738. 10.1111/bjd.13433 25272088

[5] Heelan K , Mahar A , Walsh S , Shear N . Pemphigus and associated comorbidities: a cross‐sectional study. Clin Exp Dermatol. 2015;40:593‐599. 25786337 10.1111/ced.12634

[6] Balighi K , Mahmoudi H , Tavakolpour S , Daneshpazhooh M . Coexistence of oral lichen planus and pemphigus vulgaris. Clin Oral Invest. 2018;22:1‐3. 10.1007/s00784-018-2619-3 30218230

[7] Lee CW , Hur H , Youn JI . Pemphigus vulgaris coexisting with generalized lichen planus. J Dermatol. 1987;14(4):388‐391. 3320133 10.1111/j.1346-8138.1987.tb03598.x

[8] Feng H , Gutierrez D , Rothman L , Meehan S , Sicco KL . Lichen planus pigmentosus. Dermatol Online J. 2018;24(12):13030/qt0wz1v2kd. Published 2018 Dec 15. 30677796

[9] Vachiramon V , Suchonwanit P , Thadanipon K . Bilateral linear lichen planus pigmentosus associated with hepatitis C virus infection. Case Rep Dermatol. 2010;2:169‐172. 21060775 10.1159/000320775 PMC2974975

[10] Mancuso G , Berdondini R . Coexistence of lichen planus pigmentosus and minimal change nephrotic syndrome. Eur J Dermatol. 2009;19:389‐390. 19451054 10.1684/ejd.2009.0682

[11] Sassolas B , Zagnoli A , Leroy JP , Guillet G . Lichen planus pigmentosus associated with acrokeratosis of Bazex. Clin Exp Dermatol. 1994;19(1):70‐73. 8313644 10.1111/j.1365-2230.1994.tb01122.x

[12] Robles J , Rizo‐Frías P , Herz‐Ruelas M , Pandya A , Ocampo‐Candiani J . Lichen planus pigmentosus and its variants: review and update. Int J Dermatol. 2017;57:505‐514. 29076159 10.1111/ijd.13806

[13] Bhutani LK , Bedi TR , Pandhi RK , Nayak NC . Lichen planus pigmentosus. Dermatologica. 1974;149(1):43‐50. 4154221 10.1159/000251470

[14] Rieder E , Kaplan J , Kamino H , Sanchez M , Pomeranz MK . Lichen planus pigmentosus. Dermatol Online J. 2013;19(12):20713. Published 2013 Dec 16. 24365004

[15] Ghosh A , Coondoo A . Lichen planus pigmentosus: the controversial consensus. Indian J Dermatol. 2016;61(5):482‐486. 27688435 10.4103/0019-5154.190108 PMC5029231

[16] Hong S , Shin JH , Kang HY . Two cases of lichen planus pigmentosus presenting with a linear pattern. J Korean Med Sci. 2004;19(1):152‐154. 14966361 10.3346/jkms.2004.19.1.152 PMC2822256

[17] Akarsu S , Ilknur T , Özer E , Fetil E . Lichen planus pigmentosus distributed along the lines of Blaschko. Int J Dermatol. 2013;52(2):253‐254. 21349081 10.1111/j.1365-4632.2011.04872.x

[18] Kanwar AJ , Dogra S , Handa S , Parsad D , Radotra BD . A study of 124 Indian patients with lichen planus pigmentosus. Clin Exp Dermatol. 2003;28(5):481‐485. 12950331 10.1046/j.1365-2230.2003.01367.x

[19] Neumann‐Jensen B , Worsaae N , Dabelsteen E , Ullman S . Pemphigus vulgaris and pemphigus foliaceus coexisting with oral lichen planus. Br J Dermatol. 1980;102(5):585‐590. 6992845 10.1111/j.1365-2133.1980.tb07660.x

[20] Vahide L , Zahra H , Forugh G , Nazi S . Autoantibodies to desmogleins 1 and 3 in patients with lichen planus. Arch Dermatol Res. 2017;309(7):579‐583. 28674915 10.1007/s00403-017-1756-x

[21] Mignogna MD , Fortuna G , Leuci S , Stasio L , Mezza E , Ruoppo E . Lichen planus pemphigoides, a possible example of epitope spreading. Oral Surg Oral Med Oral Pathol Oral RadiolEndod. 2010;109(6):837‐843. 10.1016/j.tripleo.2009.12.044 20382044

